# Diagnostic Accuracy of the Fluorescein Efflux Test for Congenital Nasolacrimal Duct Obstruction

**DOI:** 10.7759/cureus.81251

**Published:** 2025-03-26

**Authors:** Akiko Sawa, Futoshi Taketani, Yuki Kataoka, Chika Miyazaki

**Affiliations:** 1 Ophthalmology, Hyogo Prefectural Amagasaki General Medical Center, Amagasaki, JPN; 2 International and Community Oral Health, Tohoku University Graduate School of Dentistry, Sendai, JPN; 3 Healthcare Epidemiology, Kyoto University Graduate School of Medicine, School of Public Health, Kyoto, JPN; 4 Systematic Review, Scientific Research Works Peer Support Group, Osaka, JPN; 5 Internal Medicine, Kyoto Min-Iren Asukai Hospital, Kyoto, JPN

**Keywords:** congenital nasolacrimal duct obstruction, diagnostic accuracy study, lacrimal duct obstruction, lacrimal duct patency, pediatric patients, the fluorescein disappearance test

## Abstract

Objectives: This study aimed to assess the diagnostic accuracy of the fluorescein efflux test (FET) for congenital nasolacrimal duct obstruction (CNLDO).

Methods: We conducted a retrospective diagnostic accuracy study at a tertiary eye care center from September 2020 to December 2021. The study comprised 120 eyes from 97 patients aged <6 years suspected of CNLDO. After undergoing ophthalmologic examination, patients received both the fluorescein disappearance test (FDT) and FET. Dacryoendoscopy, syringing, and clinical findings served as reference standards. We differentiated between complete and partial lacrimal duct obstruction (LDO) by categorizing completely blocked cases as c-LDO. The primary outcome measured was the sensitivity and specificity of FET for CNLDO, with secondary outcomes focusing on sensitivity and specificity for c-LDO.

Results: Of the 120 eyes, 102 eyes qualified for CNLDO diagnosis and 102 eyes for c-LDO diagnosis. FET demonstrated a sensitivity of 77% (95% CI: 68-85) and specificity of 33% (13-59) for CNLDO. Regarding c-LDO, FET showed a sensitivity of 86% (78-92) and a specificity of 83% (59-96).

Conclusions: The diagnostic accuracy of FET was lower in patients clinically diagnosed with CNLDO compared to those with c-LDO, as some patients did not exhibit true obstruction of the distal nasolacrimal duct. FET demonstrates high diagnostic accuracy for c-LDO and may assist in diagnosing lacrimal duct patency in children. It may also help exclude conditions other than lacrimal duct obstruction in pediatric patients presenting with epiphora and eye discharge.

## Introduction

Congenital nasolacrimal duct obstruction (CNLDO) is a common condition affecting infants, characterized by epiphora or eye discharge. It arises from membranous obstruction at the distal end of the developing nasolacrimal duct [1], which typically resolves spontaneously in 82-96% of cases by the age of one year [2,3] through natural processes or conservative treatments. While other lacrimal duct disorders, such as obstruction of puncta or canaliculi, multiple blockages in the nasolacrimal duct, or hypoplasia of the lacrimal duct, may present with similar symptoms, they are less likely to resolve without intervention [4,5]. Moreover, conditions like conjunctivitis, epiblepharon, or childhood glaucoma can manifest similar symptoms even when lacrimal drainage function is normal [1,6,7], underscoring the necessity for confirmatory tests of lacrimal duct patency in symptomatic children. 

The fluorescein disappearance test (FDT) is commonly employed in children to assess lacrimal drainage function, given its non-invasive nature compared to syringing, which can be painful without anesthesia in an office setting [8]. This test involves applying fluorescein to the lower lid conjunctiva, and after 5-10 minutes, observing the tear meniscus for any remaining fluorescein. In infants with normal lacrimal drainage function, fluorescein in tears flows into the nasal passages and swiftly disappears from the tear meniscus, making FDT a simple, sensitive (90%), and specific (100%) method for diagnosing CNLDO [8]. However, FDT cannot differentiate between fluorescein reduction due to drainage into the tear duct or outflow from the eye, rendering it an indirect assessment of lacrimal duct patency.

To directly evaluate lacrimal duct patency, this study introduces the fluorescein efflux test (FET), performed simultaneously with FDT. We gently inserted separate cotton swabs into the entrance of each nostril to collect nasal discharge and observed each swab under a slit lamp with a blue-free filter. In cases of normal lacrimal ducts, fluorescein-stained tears flow into the nasal cavity, enabling the direct detection of fluorescein on the swab. This approach follows the principle of the Jones fluorescein test reported by Lester Jones in 1961 [9], which directly assesses lacrimal duct patency and demonstrates a sensitivity of 91% and a specificity of 78% in patients aged 14-85 years [10]. While the Jones fluorescein test is less commonly performed in children due to the complexity of its procedure and interpretation, FET is a simpler alternative, as it can be done alongside FDT and only requires non-invasive observation of nasal discharge within a short time, making it especially suitable for children. Nonetheless, further investigations are warranted to ascertain whether it achieves comparable diagnostic accuracy when conducted by any physician. 

In addition, we set c-LDO (complete lacrimal duct obstruction) as a target condition alongside CNLDO. There are various situations where we would like to assess lacrimal duct patency non-invasively in an outpatient setting, such as in older children who are not suitable for syringing or dacryoendoscopy due to the difficulty of keeping their body still, in patients who are not suspected of having a lacrimal disorder but need confirmation of adequate lacrimal drainage, and in those with persistent symptoms despite previous bougienage. However, at present, FDT is the only non-invasive test that can be routinely performed in children. Since it requires subjective evaluation, interpretation can sometimes be challenging. Therefore, by clarifying the diagnostic accuracy of FET for c-LDO, we aim to objectively assess lacrimal duct patency in pediatric patients beyond CNLDO and demonstrate that FET can complement or enhance FDT. The primary objective of this study was to assess the diagnostic accuracy of FET for CNLDO. We also diagnosed c-LDO and secondarily evaluated the diagnostic accuracy of FET for c-LDO.

## Materials and methods

Our diagnostic accuracy study complies with the STARD 2015 guidelines (Figures 4, 5, 6 of appendices). This retrospective investigation was conducted in accordance with the Declaration of Helsinki at the Ophthalmology Department of the Hyogo Prefectural Amagasaki General Medical Center (AGMC) between September 2020 and December 2021. Annually, the Ophthalmology Department at AGMC manages approximately 850 patients with lacrimal duct disorders. The Ethics Committee of AGMC approved the study protocol and waived the requirement for written informed consent due to the retrospective nature of the study. The ethical approval number was 3-160.

The study encompassed 120 eyes from 97 consecutive patients under the age of six years, who were referred by their family physicians after receiving conservative treatments for suspected CNLDO and initially presented at the Ophthalmology Department of AGMC between September 2020 and December 2021. To mirror real-world clinical scenarios, we included consecutive cases without exclusion criteria, such as chromosomal abnormalities, prior surgical interventions, punctal stenosis, or external eye disorders like epiblepharon.

Following interviews and examinations, we conducted FDT before FET. Fluorescein was applied to the bilateral lower eyelid margins and evaluated after 5-10 minutes. A positive FDT result was defined as residual fluorescein at the eyelid margin, indicating lacrimal duct obstruction. Cases in which the eyes were filled with tears and fluorescein flowed out of the ocular surface were classified as "ungradable."

Index test

We performed FET as the index test. Using fluorescein eye test paper (FLUORES Ocular Examination Test Paper 0.7 mg®; AYUMI Pharmaceutical Corporation, Tokyo, Japan) and a single drop of artificial tear ophthalmic solution (Artificial Tear Mytear Ophthalmic Solution®; SENJU Pharmaceutical Corporation, Osaka, Japan), we applied these to the bilateral tear menisci at the lower eyelid margins of each patient. After 5-10 minutes, cotton swabs (7.8 × 0.5 cm) were inserted into the entrance of each nostril to collect nasal discharge. Subsequently, we examined these swabs under a slit-lamp microscope equipped with a blue-free filter (SLIT LAMP SL-D701; Topcon Corporation, Tokyo, Japan) to detect fluorescence. In this study, nasal discharge was collected immediately after evaluating the FDT results, as the tears had already been stained with fluorescein during the FDT procedure.

**Figure 1:**
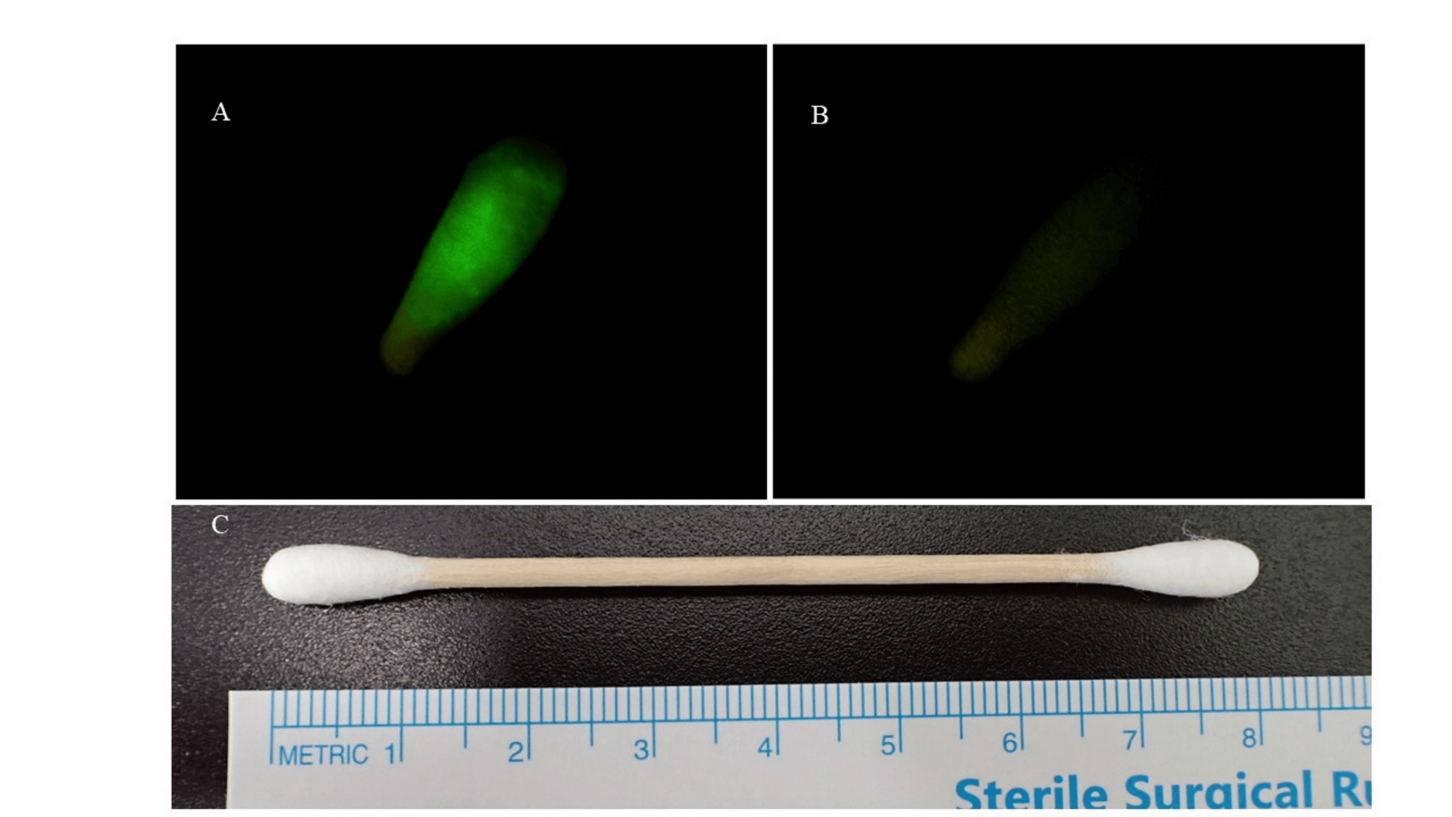
The FET results A: It indicates a negative FET result, suggesting no lacrimal duct obstruction. B: It indicates a positive FET result, potentially suggesting lacrimal duct obstruction. No fluorescence is emitted, so the screen appears dark. C. It indicates a cotton swab used in our study. FET, fluorescein efflux test

Fluorescein has a maximum absorption wavelength of 494 nm and a maximum emission wavelength of 521 nm. The use of a slit-lamp microscope with a blue-free filter (approximately 530 nm) enhanced fluorescence contrast and facilitated detection. If fluorescence was observed, indicating the presence of fluorescein in the tear that had flowed into the nasal cavity via the lacrimal duct, it suggested no lacrimal duct obstruction, and we considered the FET result negative (Figure 1A). Conversely, if fluorescence was not observed, suggesting a potential lacrimal duct obstruction, the sample was considered FET positive (Figure 1B).

A single physician administered both FDT and FET.

Reference standard

The target conditions for this study were CNLDO and c-LDO. For CNLDO cases, the diagnosis was confirmed as part of the treatment (probing) through dacryoendoscopy or syringing, which identified an obstruction at the distal end of the nasolacrimal duct. Dacryoendoscopy was basically used as the reference standard unless the parents opted for follow-up observation. Syringing was performed only if they declined dacryoendoscopy. When the patient was younger than six months or preferred observation over immediate treatment, the diagnosis was made based on specific clinical findings. In c-LDO cases, the diagnosis was made when visible punctal obstruction was observed, or when complete obstructions in the lacrimal duct were identified through dacryoendoscopy. Additionally, cases diagnosed as CNLDO based on observation or syringing at the request of the guardians were also included. Dacryoendoscopy, syringing, and clinical findings were used as reference standards, all performed by several physicians specializing in the treatment of lacrimal duct disorders. We believe that these results reflect the true status of CNLDO and c-LDO as diagnostic targets.

Dacryoendoscopy was conducted under topical or general anesthesia, allowing for the observation of not only membranous closure at the distal end of the nasolacrimal duct but also various changes such as lacrimal calculi. Additionally, procedures such as probing or stent placement could be performed simultaneously [11-13].

Syringing was performed using a Bangerter irrigation needle (18G). This procedure is commonly used to evaluate lacrimal duct patency in adults and can also confirm patency in CNLDO patients while allowing simultaneous probing.

Clinical findings were obtained through medical history interviews and observation of characteristic symptoms, including epiphora, sticky eyes with mucous discharge, reverse flow of secretions from the lacrimal sac, height of the tear meniscus, and positive FDT results. This procedure aligns with the diagnostic criteria for CNLDO available on the AAPOS website (https://www.aapos.org/glossary/nasolacrimal-duct-obstruction).

We documented cases where parents chose observation at the initial visit to account for potential bias. Cases where FET and the reference standard were conducted by the same physician were also documented to consider the potential impact of the FET result on the reference standard. While we usually conducted the reference standard immediately following the FET, cases requiring general anesthesia were scheduled promptly, and the number of such cases was documented. Additionally, cases with findings other than CNLDO, such as abnormal puncta and epiblepharon, which could cause epiphora, were documented and included in this study, considering the possibility of concurrent CNLDO.

Outcomes

Primary Outcome

The primary outcome assessed the sensitivity and specificity of FET in comparison with the reference standard for CNLDO.

Secondary Outcome

The secondary outcome evaluated the sensitivity and specificity of FET compared to the reference standard for c-LDO.

Statistical analyses

Our statistical analyses were conducted using RStudio Cloud, adhering to a predefined statistical analysis plan. Initially, we summarized the characteristics of the participants. Cross-tabulations of the index test against the reference standard were performed, and sensitivity, specificity, along confidence intervals, were calculated to estimate the precision of the diagnostic accuracy measures. Furthermore, sensitivity analyses were undertaken by excluding observational cases or instances where the same physician conducted both the index test and the reference standard.

Sample size

The determination of the sample size was based on the number of cases observed in our hospital during the study period.

## Results

Participant characteristics

**Figure 2:**
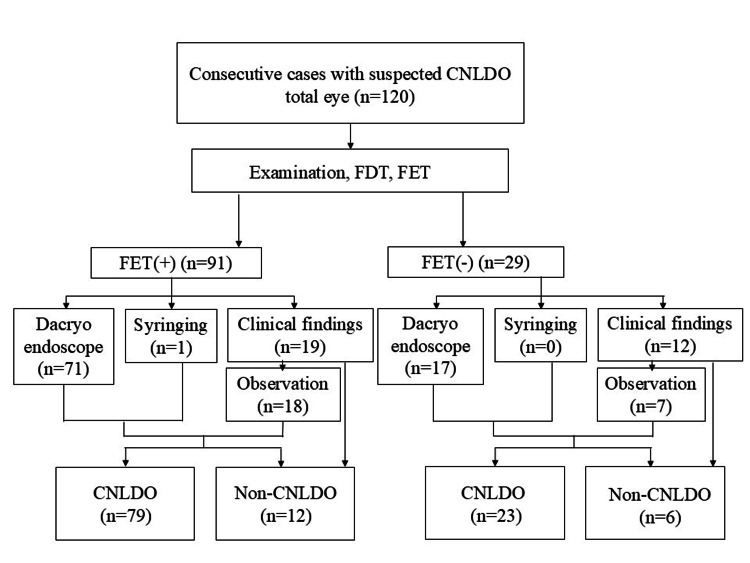
Flow of participants in the diagnosis of CNLDO CNLDO, congenital nasolacrimal duct obstruction; FDT, fluorescein disappearance test; FET, fluorescein efflux test

**Figure 3:**
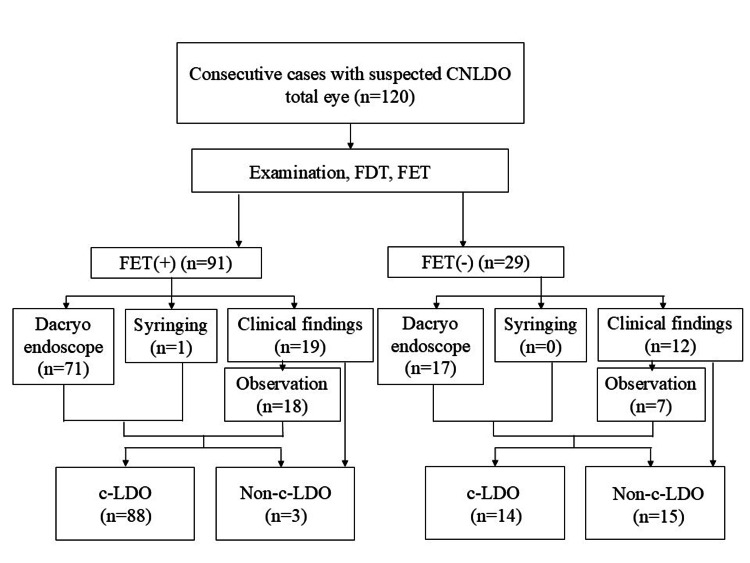
Flow of participants in the diagnosis of c-LDO CNLDO, congenital nasolacrimal duct obstruction; FDT, fluorescein disappearance test; FET, fluorescein efflux test; c-LDO, complete lacrimal duct obstruction

**Figure 4:**
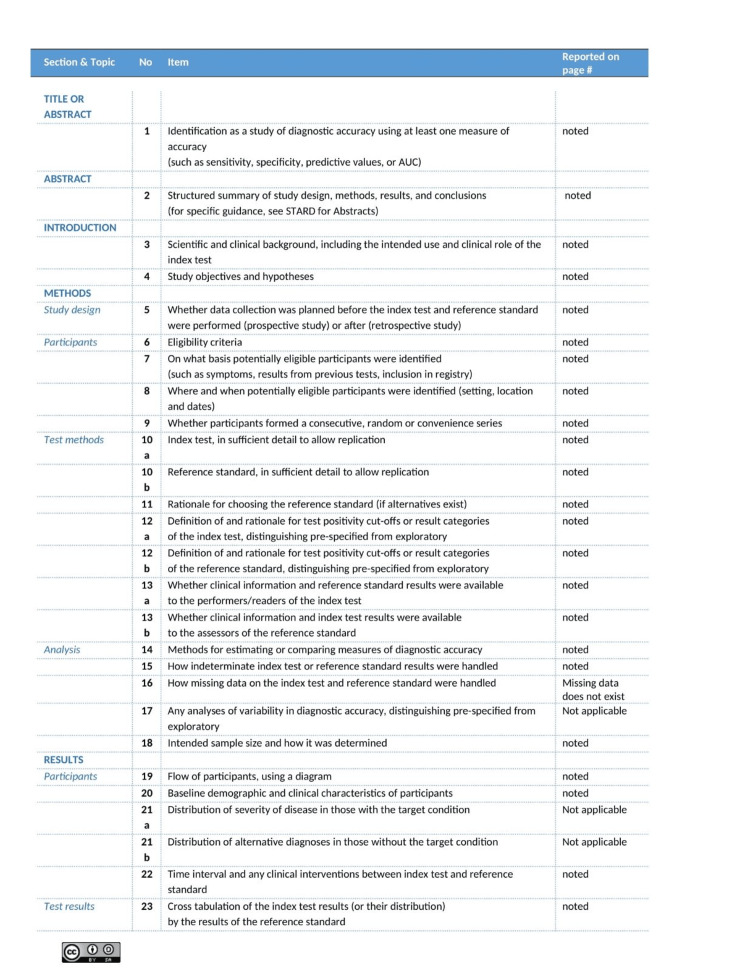
Our study meets the STARD 2015 guidelines, Page 1.

**Figure 5:**
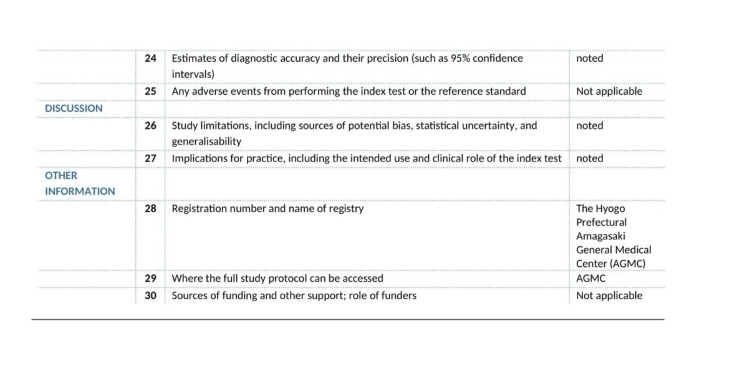
Our study meets the STARD 2015 guidelines, Page 2.

**Figure 6:**
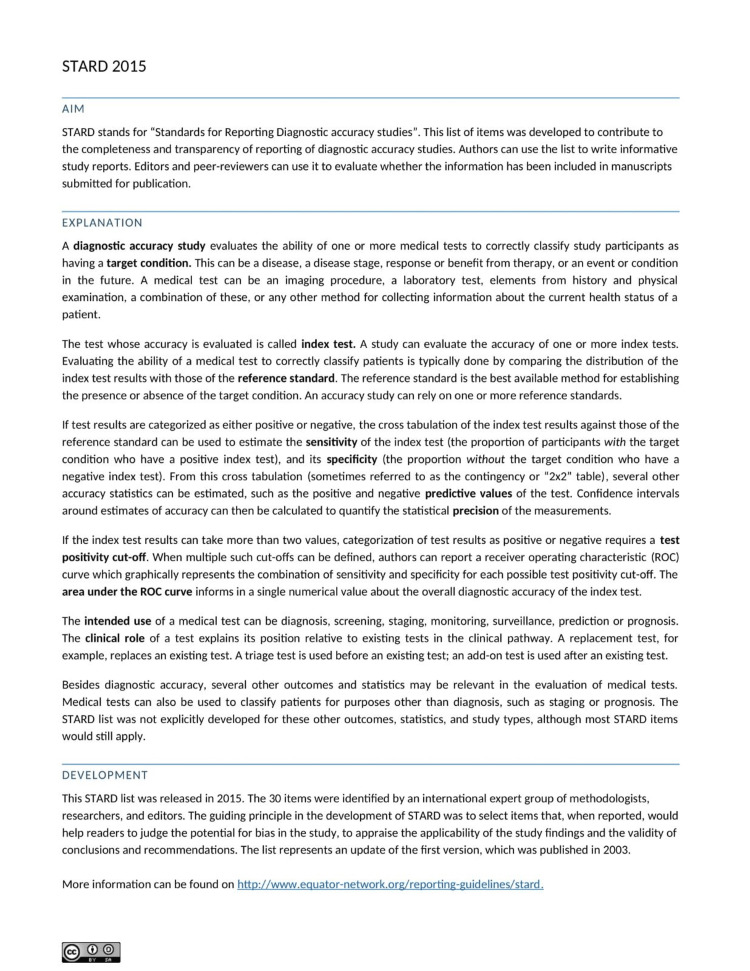
Our study meets the STARD 2015 guidelines, Page 3.

We describe the participant flow in this study. Out of 120 eyes, 91 tested positive and 29 tested negative in FET (Figures 2, 3).

Table 1 presents the characteristics of the study population.

**Table 1:** Participant characteristics (n=120) CNLDO, congenital nasolacrimal duct obstruction; c-LDO, complete lacrimal duct obstruction

Characteristics	n (%, n/120)
Age (month), median (IQR)	12 (9-17)
Male gender	66 (55)
CNLDO	102 (85)
Complete obstruction	92
Partial obstruction	10
Non-CNLDO	18 (15)
No lacrimal duct obstruction with Down syndrome	2
Multiple lacrimal duct obstruction with Down syndrome	2
Iatrogenic obstruction	2
Iatrogenic stenosis	1
Punctal obstruction	5
Punctal stenosis	2
Conjunctivitis with punctal stenosis	1
Epiblepharon, no lacrimal duct obstruction	2
Lacrimal duct obstruction with swelling over the medial canthus due to EB virus	1
c-LDO	102 (85)
Observation	25 (21)
Reference standard with: dacryoendoscope	88 (73)
Syringing	1 (1)
Clinical findings	31 (26)
The same doctor did the index test and the reference standard	21 (18)
Dacryoendoscope could not be performed immediately after the index test	14 (12)

We diagnosed CNLDO in 102 out of 120 eyes, with 92 eyes demonstrating c-LDO and 10 eyes showing partial obstruction. Of 120 eyes, 102 eyes were diagnosed with c-LDO, comprising 92 eyes with CNLDO and 10 eyes without CNLDO. In Figure 3, out of 91 FET-positive cases, 88 were diagnosed as c-LDO. Among them, 69 cases were diagnosed using dacryoendoscopy, one case by syringing, and 18 cases based on clinical findings. Of these 18 cases, five had punctal occlusion, one had lacrimal sac swelling and dacryocystitis due to EB virus infection, and two were later confirmed to have obstructions at the distal end of the nasolacrimal duct when undergoing probing with dacryoendoscopy. The remaining 10 cases were diagnosed as CNLDO based on FDT positivity and typical symptoms of CNLDO, as their guardians opted for observation. These cases were classified as c-LDO. We observed 25 eyes, of which 20 were diagnosed with CNLDO based on clinical findings during their initial visit. The reference standard included 88 eyes diagnosed using dacryoendoscopy, one eye through syringing, and 31 eyes based solely on clinical findings. In 21 eyes, the same physician conducted both the index test and reference standard. In 14 eyes, dacryoendoscopy was not feasible in the office setting, leading to the scheduling for general anesthesia at a later date.

Diagnostic accuracy

Table 2 presents the sensitivity and specificity of FET for CNLDO and c-LDO.

**Table 2:** Sensitivity and specificity of FET for diagnosing CNLDO and c-LDO FET, fluorescein efflux test; CNLDO, congenital nasolacrimal duct obstruction; c-LDO, complete lacrimal duct obstruction

Target condition	Sensitivity (%) (95% CI)	Specificity (%) (95% CI)
CNLDO	77 (68-85)	33 (13–59)
c-LDO	86 (78-92)	83 (59-96)

The sensitivity and specificity of FDT could not be evaluated in this study due to the considerable number of ungradable cases (Tables 3, 4). 

**Table 3:** Cross tabulations of FDT results versus the presence of c-LDO FDT, fluorescein disappearance test; c-LDO, complete lacrimal duct obstruction

		c-LDO (+)	c-LDO (-)	Total
FDT	Positive	52	3	55
	Negative	8	9	17
	Ungradable	42	6	48
	Total	102	18	120

**Table 4:** Cross tabulations of FDT results versus presence of CNLDO FDT, fluorescein disappearance test; CNLDO, congenital nasolacrimal duct obstruction

		CNLDO (+)	CNLDO (-)	Total
FDT	Positive	49	6	55
	Negative	12	5	17
	Ungradable	41	7	48
	Total	102	18	120

To address potential bias, we conducted a sensitivity analysis (Table 5).

**Table 5:** Sensitivity analysis of FET FET, fluorescein efflux test; CNLDO, congenital nasolacrimal duct obstruction; c-LDO, complete lacrimal duct obstruction

Target condition	Sensitivity (%) (95% CI)	Specificity (%) (95% CI)
Excluding observation cases		
CNLDO	80 (69-88)	38 (15-65)
c-LDO	85 (76-92)	77 (46-95)
Excluding cases where the index test and the reference standard were conducted by the same doctor		
CNLDO	80 (70-88)	29 (8-58)
c-LDO	88 (79-94)	79 (49-95)

## Discussion

In this investigation, we assessed the sensitivity and specificity of FET as an index test for CNLDO and c-LDO. Our findings revealed that FET exhibited greater sensitivity and specificity for c-LDO compared to CNLDO, notably improving specificity. Furthermore, sensitivity analyses were conducted and confirmed the robustness of the diagnostic accuracy of FET for both CNLDO and c-LDO.

Our results indicate that FET enables the objective assessment of lacrimal duct patency in children in a minimally invasive and visually comprehensible manner. The diagnostic accuracy of FET for CNLDO in our study was lower than that of FDT reported by Young (sensitivity 90%, specificity 100%) [8]. However, not all studies have reported such high accuracy. For example, a study published in 2001 reported FDT sensitivity and specificity as 76%/76% at 5 minutes and 63%/89% at 10 minutes [14]. Both FDT and FET can exhibit variability in accuracy depending on testing methods and conditions. In our study, the diagnostic accuracy of FDT could not be determined because many patients cried, causing fluorescein to flow out of their eyes, which made evaluation impossible. On the other hand, when patients cry, fluorescein flows more easily into the nose, making FET more useful for evaluation when FDT is inconclusive. FDT and FET can complement each other, and performing them simultaneously has advantages. The key difference between FET and FDT is that FET provides an objective evaluation, where detecting fluorescence is a simple binary decision. In contrast, FDT relies on a qualitative assessment of fluorescein retention in the tear meniscus, which can vary between examiners.

We reclassified cases between CNLDO and c-LDO as target conditions, and the sensitivity and specificity of FET for c-LDO became higher than for CNLDO. Among the 91 FET-positive cases, 79 were diagnosed as CNLDO and 12 as non-CNLDO. Of these 12 cases, nine were classified as c-LDO(+), leading to higher specificity due to a decrease in false positives. These nine cases included five with punctal occlusion, two with multiple LDOs associated with Down syndrome, and two with iatrogenic obstruction due to past bougie treatment. Additionally, among the 29 FET-negative cases, 23 were diagnosed as CNLDO and six as non-CNLDO. Of the 23 CNLDO cases, nine were reclassified as c-LDO(-), resulting in higher sensitivity due to a decrease in false negatives. These nine cases included two who showed good irrigation during surgery for epiblepharon, with postoperative symptom improvement; 1 with conjunctivitis and discharge who was initially FDT-positive but showed symptom resolution after treatment; one without typical symptoms such as discharge but with ungradable FDT results, whose symptoms quickly improved with observation; four with dacryolithiasis but no distal nasolacrimal duct obstruction; and one with incomplete opening of the lower nasolacrimal duct observed on dacryoendoscopy. The low specificity of FET in the CNLDO group may lead to overdiagnosis. However, the false-positive cases included those with Down syndrome, iatrogenic lacrimal obstruction, and punctal occlusion, all of which require treatment. High specificity for c-LDO leads to a lower risk of overtreatment. Conversely, a negative FET result strongly suggests the absence of lacrimal obstruction, providing a good opportunity to actively consider other possible diseases.

In cases of false positives on the FET, additional examinations such as dacryoendoscopy or lacrimal syringing are performed to confirm patency. The benefit is that if obstruction or stenosis is present, treatment options such as stent placement can be carried out during the same procedure [15]. If no lacrimal duct disorder is found, other potential causes, such as epiblepharon, can be considered [1], and appropriate treatment can be initiated. Performing dacyoendoscopy or syringing when the lacrimal system is not the cause does not present significant long-term risks.

FET may help avoid unnecessary invasive procedures. While CNLDO is often diagnosed based on history and symptoms [1], in atypical cases, such as those with persistent symptoms beyond the age of two or those that present after six months of age, confirming lacrimal patency before invasive intervention is crucial. FDT is useful, but results may vary between examiners due to visually checking the fluorescein remaining in the tear meniscus [8,16,17]. In contrast, FET provides a direct evaluation by visualizing fluorescein in nasal discharge, offering objective results. Combining these different diagnostic approaches can improve accuracy. This aligns with the goal of evaluating lacrimal duct patency in children to avoid unnecessary invasive tests or treatments in patients without lacrimal duct disease. 

Family physicians frequently see pediatric patients presenting with epiphora or eye discharge, but the available tests that can be performed in an outpatient setting are limited. Performing FET alongside FDT allows for a more accurate evaluation of lacrimal patency in that situation, enabling more appropriate probing for CNLDO cases that do not resolve with conservative treatments. Even in cases with insufficient improvement after probing, FET can assess patency without the need for invasive irrigation. FET is a simple procedure that can be performed by any physician, including nonspecialists.

There are limitations in our study. Many FDT outcomes were ungradable due to adherence to our protocol, making it difficult to compare FDT and FET results. In Japan, parents are usually asked to wait in the waiting room during surgical procedures or probing on their children to reduce their mental stress, and the same approach was taken in our study. Consequently, this led to excessive crying in patients, which in turn made it difficult to evaluate the FDT results. Furthermore, among the 91 FET-positive cases, 88 were diagnosed as c-LDO. Of these, 11 were classified as CNLDO based on syringing or clinical findings (one by syringing, 10 by clinical findings and FDT), and one case was diagnosed with dacryocystitis due to EB virus infection. Since the parents chose observation, dacryoendoscopic confirmation was not possible, leaving a slight possibility that some of these 12 cases were not strictly c-LDO. In addition, fluorescein reflux from the opposite nasal cavity may cause false negatives despite LDO, potentially diminishing FET sensitivity and underestimating its diagnostic accuracy. Similarly, anatomical factors may impair nasal discharge collection, leading to false positives that lower specificity and further contribute to the underestimation of its diagnostic accuracy. Therefore, future research is needed to validate the diagnostic accuracy of FET, particularly for LDO, across physicians with different specialties and to determine the appropriate cases for its use.

## Conclusions

In summary, FET emerges as a valuable diagnostic modality for evaluating CNLDO and c-LDO. This study underscores the need for further research to ascertain whether FET consistently delivers comparable diagnostic accuracy regardless of the expertise level of individual physicians.
